# RecA-dependent programmable endonuclease Ref cleaves DNA in two distinct steps

**DOI:** 10.1093/nar/gkt1342

**Published:** 2013-12-26

**Authors:** Erin A. Ronayne, Michael M. Cox

**Affiliations:** Department of Biochemistry, University of Wisconsin-Madison, Madison, WI 53706, USA

## Abstract

The bacteriophage P1 recombination enhancement function (Ref) protein is a RecA-dependent programmable endonuclease. Ref targets displacement loops formed when an oligonucleotide is bound by a RecA filament and invades homologous double-stranded DNA sequences. Mechanistic details of this reaction have been explored, revealing that (i) Ref is nickase, cleaving the two target strands of a displacement loop sequentially, (ii) the two strands are cleaved in a prescribed order, with the paired strand cut first and (iii) the two cleavage events have different requirements. Cutting the paired strand is rapid, does not require RecA-mediated ATP hydrolysis and is promoted even by Ref active site variant H153A. The displaced strand is cleaved much more slowly, requires RecA-mediated ATP hydrolysis and does not occur with Ref H153A. The two cleavage events are also affected differently by solution conditions. We postulate that the second cleavage (displaced strand) is limited by some activity of RecA protein.

## INTRODUCTION

Recombination enhancement function (Ref) is a 21-kDa RecA-dependent endonuclease encoded by the temperate bacteriophage P1 (1). *In vivo*, expression of Ref produces an enhancement of multiple classes of recombination events in a RecA-dependent manner (2–4). The function of *ref* in the phage P1 life cycle is unknown, as *Δref* phage is able to replicate normally in both the lytic and lysogenic modes (2). However, transcription of *ref* is under the control of the phage P1 c1 master repressor, suggesting that *ref* transcription plays a role in the lytic cycle (5). This repressor functions in an autoregulatory control loop, binding 22 sites on the P1 genome. It maintains the lysogenic replication cycle of P1 by blocking the transcription of genes necessary for lytic induction (6). Upon phage infection, the linear double-stranded P1 genome must be cyclized to avoid degradation by host exonucleases. This process occurs either by RecA-dependent homologous recombination of terminally redundant ends, or by phage-encoded Cre-*lox* site-specific recombination (6). Ref may play a role in RecA-dependent cyclization, and thus the maintenance of P1 as a low copy number plasmid (7).

More recently, the biochemical activity of Ref as a RecA-dependent endonuclease was elucidated. Thus, Ref represents both a new class of enzyme and a new function for the RecA protein (1). Ref binds to single-stranded DNA (ssDNA) and double-stranded DNA (dsDNA) in the absence of cofactors or other proteins. Ref alone is not able to degrade DNA. However, when RecA, ATP and Mg^2+^ are included in the presence of circular ssDNA (cssDNA), Ref cleaves the cssDNA. This activity of Ref is dependent on the presence of active RecA nucleoprotein filaments on the ssDNA, which require Mg^2+^ and ATP to form. No sequence specificity has been identified for Ref-mediated DNA cleavage. Use of an ATPase-deficient RecA variant K72R, or the minimally hydrolyzable ATP analog ATPγS, abolishes Ref endonuclease activity on cssDNA.

Ref may access the DNA by binding in the RecA filament groove. Ref activity *in vitro* is blocked by a non-hydrolyzable LexA variant, a protein known to bind tightly in the RecA filament groove (1). The ability of Ref to enhance RecA-dependent recombination *in vivo* in *Escherichia coli* P1 lysogens is not affected by *lexA3* mutants, which do not undergo the bacterial SOS response (3,8).

The 3D structure of the C-terminal domain of Ref, including the nuclease active site, has been determined to 1.4-Å resolution (PDB ID: 3PLW) (1). The asymmetric unit contains a Ref monomer stably bound to two Zn^2+^ ions. Although the crystallized protein is full-length, the N-terminal 76 amino acids are disordered. This N-terminal region is highly charged with residues contributing 25 positive and 9 negative charges, making up 45% of the 76 amino acids in total. The N-terminal region is required for DNA binding, and thus no DNA binding motif is included in the solved structure (1).

Structural analysis places Ref in the H-N-H family of homing endonucleases, which includes several other bacteriophage-encoded proteins. This family is defined by a ββα metal-binding core. However, outside this core, there is little structural or functional similarity among the members of this protein family (9). In Ref, this motif is made up of a two-stranded β-hairpin between several α-helices. One of the metal binding sites is the solvent-exposed active site, with the Zn^2+^ ion tetrahedrally coordinated to a sulfate ion and three histidine residues. The sulfate ion is likely in the same position as the scissile phosphate from the DNA backbone. In addition, when alanine is substituted for His 153, which coordinates this Zn^2+^ ion, Ref still folds properly. However, all nuclease activity on cssDNA is lost, speaking to the importance of His 153 in catalysis (1).

Most interestingly, and the focus of this study, the endonuclease activity of Ref can be targeted to displacement loops (D-loops). D-loops are created when RecA-bound oligonucleotides (oligos) invade homologous regions of duplex DNA. This strand invasion creates heteroduplex DNA, with one strand of the original duplex being displaced by the RecA-bound oligo. On the addition of Ref, either one or both of the original duplex strands is cleaved, generating nicked circular dsDNA (cdsDNA) or linear dsDNA (1). We refer to this as the targeted nuclease activity of Ref.

Here, we report on the mechanism of RecA nucleoprotein filament-targeted double-strand break (DSB) generation by the Ref protein within D-loops of homologous duplex DNA. This reaction has the potential for use in genomic engineering. Recently, the CRISPR/Cas system has been investigated in a similar context. Cas9 endonuclease targets R-loops *in vivo*, using two different active sites for cleavage: an HNH site for the paired strand of the R-loop and an RuvC-like domain for the displaced strand (10). Ref functions similarly as a targeting endonuclease, but uses DNA oligonucleotides to guide the reaction as opposed to RNA oligonucleotides. Before the Ref protein can be fully used, more must be understood about the mechanism of RecA filament-guided cleavage.

## MATERIALS AND METHODS

### Proteins

The *E. coli* RecA E38K protein was purified as described previously (11), with the following modifications. After washing the protein pellet with R buffer plus 2.1 M ammonium sulfate, the pellet was resuspended in R buffer plus 1 M ammonium sulfate. The sample was loaded onto a butyl-Sepharose column and washed with 2 column volumes of R buffer plus 1 M ammonium sulfate. It was then eluted with a linear gradient from R buffer plus 1 M ammonium sulfate to R buffer, carried out over 10 column volumes. Peak fractions were identified by sodium dodecyl sulphate-polyacrylamide gel electrophoresis analysis and pooled. The protein was loaded onto a hydroxyapatite column as done previously, but with the linear gradient from 20 to 500 mM P buffer. The fractions were dialyzed against R buffer and loaded on to a Source 15Q column. The column was washed with 2 column volumes of R buffer, and protein was eluted with a linear gradient from no salt to 1 M KCl, again carried out over 10-column volumes. Peak fractions were identified as above, pooled and dialyzed against R buffer. The pooled fractions were loaded onto a DEAE-Sepharose column, washed with 2 column volumes R buffer and eluted with a linear gradient from R buffer to R buffer plus 1 M KCl over 10 column volumes. The peak fractions were identified and pooled as above, precipitated with ammonium sulfate (45% final concentration) and resuspended in R buffer plus 1 M KCl. The protein was loaded on an S-300 sizing column, washed with 1.5 column volumes R buffer plus 1 M KCl and fractions were identified and pooled as above. The protein was precipitated with ammonium sulfate as above, and resuspended in R buffer plus 1 M ammonium sulfate. The protein was loaded on a butyl-Sepharose column and eluted in a 15 column volume linear gradient to R buffer. The fractions were identified, pooled, precipitated as above and dialyzed against R buffer. The protein was flash-frozen in liquid nitrogen and stored at −80°C. The RecA K72R protein was purified as described previously (12). The concentration of the purified RecA protein was determined from the absorbance at 280 nm using the extinction coefficients of 2.23 × 10^4^ M^−^^1^ cm^−^^1^ (13). The Ref and Ref H153A proteins were purified as described previously (1), and concentration was determined using the published extinction coefficient of 2.851 × 10^4^ M^−^^1^ cm^−^^1^. Low-level RecA-independent duplex nicking activity was detected in the wild-type (WT) Ref nuclease preparation. This is found in most preps, and we do not know whether it is an inherent activity of Ref. No exonuclease or other endonuclease activities were detected in a detailed screen of nuclease activities.

### DNA substrates

The M13mp18 cdsDNA was prepared as described previously (14–16). The M13mp18 nicked dsDNA substrate was prepared by nicking with Nb.BsmI according to manufacturer’s recommendations. All DNA concentrations are given in terms of total nucleotides. Oligonucleotides were purchased from Integrated DNA Technologies. Sequences of oligonucleotides used in this study are presented in Table 1.

**Table 1.** Primers and oligonucleotides used in this study

Name	Length (nt)	Sequence	Complementary to M13mp18 at bases
rlb1	150	ttttggtttttatcgtcgtctggtaaacgagggttatgatagtgttgctcttactatgcctcgtaat tccttttggcgttatgtatctgcattagttgaatgtggtattcctaaatctcaactgatgaa tctttctacctgtaataatgt	597–746
5' FAM-rlb1 100	100	FAM-ttttggtttttatcgtcgtctggtaaacgagggttatgatagtgttgctcttactatgcct cgtaattccttttggcgttatgtatctgcattagttgaa	597–696
mcg3	150	tcccgactggaaagcgggcagtgagcgcaacgcaattaatgtgagttagctcactcattag gcaccccaggctttacactttatgcttccggctcgtatgttgtgtggaattgtgagcgg ataacaatttcacacaggaaacagctatga	6063–6212
mcg3 rev	150	tcatagctgtttcctgtgtgaaattgttatccgctcacaattccacacaacatacgagccgga agcataaagtgtaaagcctggggtgcctaatgagtgagctaactcacattaattgc gttgcgctcactgcccgctttccagtcggga	6212–6063 (minus strand)
5' FAM-mcg3 100 nt	100	FAM-tcccgactggaaagcgggcagtgagcgcaacgcaattaatgtgagttagctcac tcattaggcaccccaggctttacactttatgcttccggctcgtatg	6063–6162

FAM, 6-carboxyfluorescein.

### Site-specific nuclease targeting assay

Unless noted otherwise, the reactions were carried out at 37°C in buffer A*(8.5, 15), where 8.5 corresponds to the pH and 15 corresponds to the Mg^2+^ ion concentration in mM. This buffer contained 25 mM Tris-acetate (pH 8.5), 1 mM dithiothreitol, 3 mM potassium glutamate, 15 mM magnesium acetate and 5% w/v glycerol. Reactions also included an ATP regeneration system (10 U/ml pyruvate kinase and 3.5 mM phosphoenolpyruvate. The above components were incubated for 10 min with a targeting oligonucleotide (4 µMnt, either rlb1 or mcg3) and RecA E38K or RecA K72R (1.33 µM). ATP or ATPγS (3 mM) was added and incubated for additional 20 min, followed by M13mp18 cdsDNA (8 µMnt) and another 20-min incubation. Before adding Ref, a 0 time point was taken, and then Ref (100 nM) was added. The reactions were stopped at the noted time points by removing 20 µL from the reaction and adding it to 20 µl of stop solution [12 mM Tris-acetate, pH 7.5, 10.8% (w/v) Ficoll, 0.15% (w/v) each bromophenol blue and xylene cyanol, 8% sodium dodecyl sulphate] and incubating at 37°C for additional 30 min. Samples were analyzed by electrophoresis on a 0.8% agarose gel with Tris-acetate + ethylenediaminetetraacetic acid buffer, stained with SYBR Gold nucleic acid gel stain (Invitrogen) and imaged using the SYBR gold settings on a Typhoon variable mode imager (GE Healthcare). Gel image was analyzed using ImageQuantTL software (GE Healthcare). Lanes were normalized for loading conditions by reporting individual band intensity as a percentage of the total band intensity in that lane.

### Site-specific nuclease targeting assay to determine strand preference

Reactions were carried out as described using either the mcg3 or mcg3 rev targeting oligonucleotides and M13mp18 dsDNA that had been nicked with Nb.BsmI. Time points (20 µl) from the reactions were stopped with 2 µl of 0.5 M ethylenediaminetetraacetic acid (EDTA) and incubated an additional 30 min. Ten microliters of 2× alkaline loading dye [100 mM NaOH, 40 mM EDTA, 5% (w/v) Ficoll and 0.15% xylene cyanol] was added to 10 µl of the stopped reaction. An alkaline agarose gel was prepared by dissolving 0.8% (w/v) agarose in gel prep buffer (50 mM NaCl, 1 mM EDTA). Once solidified, the gel was soaked in running buffer (50 mM NaOH, 1 mM EDTA) for 30 min. Samples were run, and the gel was neutralized and deionized in water overnight and stained and imaged as above.

### Site-specific nuclease targeting assay to determine oligonucleotide cut sites

Reactions were carried out as originally noted, except 5′-carboxyfluorescein-labeled oligonucleotides were used for targeting in a total reaction volume of 30 µl. The entire reaction was stopped after 3 h with a phenol/chloroform/isoamyl alcohol extraction, followed by an ethanol precipitation. The pellets were resuspended in 8 µl of 95% formamide, 25 mM EDTA, heated at 95°C for 10 min and then quick-cooled in an ice water bath for 10 min. The samples were loaded on a 12% denaturing acrylamide sequencing gel and run at 30 W for ∼3 h in 1× Tris-borate + EDTA buffer. The gel was imaged using FAM settings on the Typhoon variable mode imager.

## RESULTS

### Ref creates targeted DSBs by cutting the paired strand of the D-loop first, followed by cutting the displaced strand

**Figure 1.**
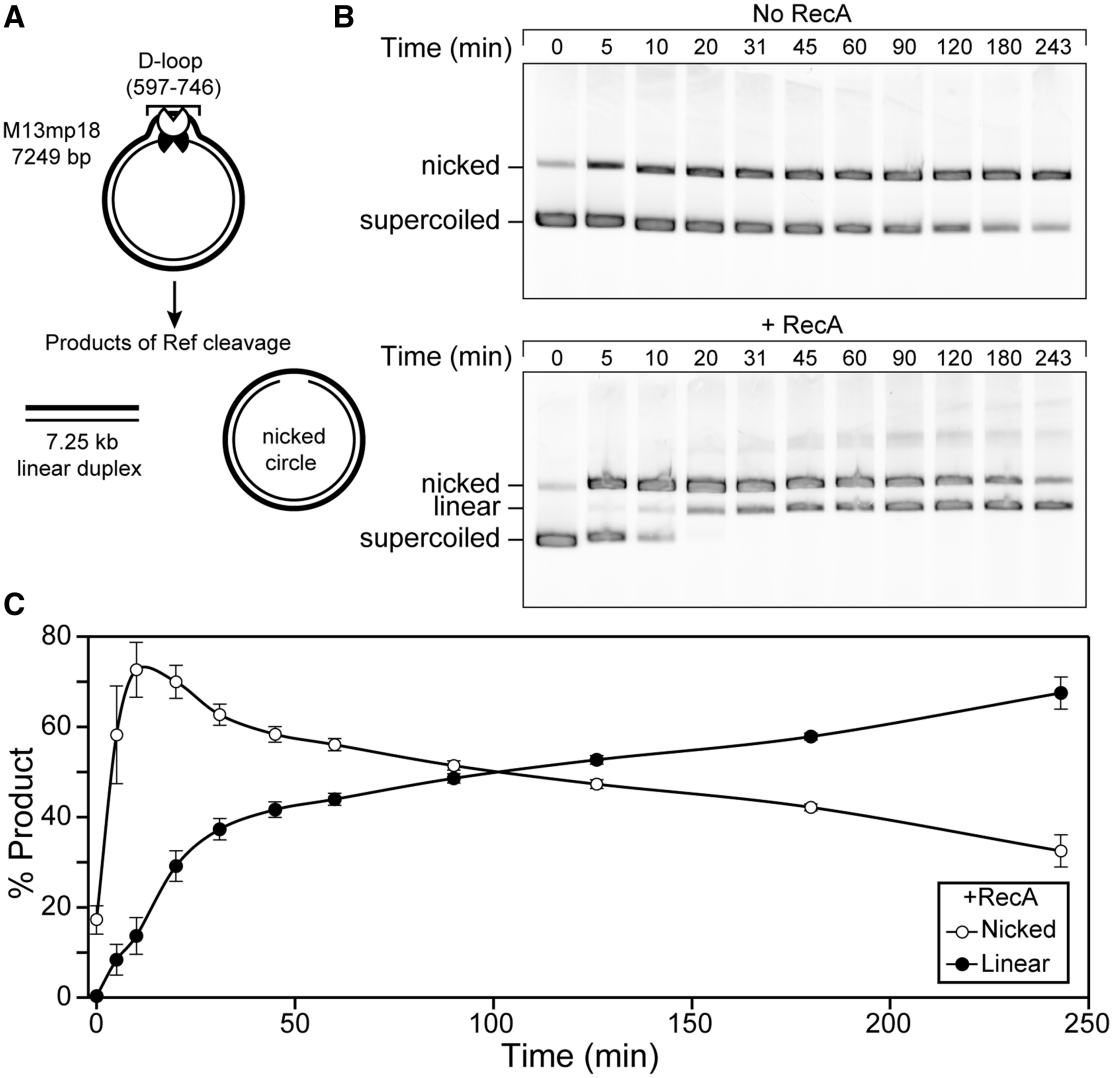
Ref creates targeted DSBs in cdsDNA through a nicked intermediate. (**A**) The reaction scheme is shown. RecA E38K (1.33 µM) was incubated with rlb1 oligonucleotide (4 µMnt) in buffer A* (8.5, 15) with an ATP regeneration system for 10 min to form a RecA filament. ATP (3 mM) was added and incubated for 20 min, followed by the addition of M13mp18 cdsDNA (8 µMnt). This was incubated for 20 min and a D-loop structure formed at positions 597–746 in the dsDNA (shown). Ref (100 nM) was added, and DNA was cleaved within the D-loop. One nick by Ref results in nicked cdsDNA, whereas two cuts result in linear dsDNA. (**B**) Agarose gel representing time points from the targeted nuclease assay, without and with (top and bottom, respectively) RecA E38K present. Supercoiled DNA is the starting material (bottom band), which is converted first to nicked DNA (top band) and subsequently to linear DNA (middle band). A small amount of RecA-independent nuclease activity is seen (top). (C)*.* Graphical representation of (B; bottom) averaged over three independent experiments. Nicked product (open circles) appears quickly, and then disappears as it is converted to linear product (closed circles).

Ref endonuclease activity can be restricted to D-loops created by the RecA protein (1). When a D-loop is created in supercoiled dsDNA, Ref cleaves one strand of the D-loop, resulting in nicked cdsDNA, or both strands, creating a 7.25-kb linear duplex (Figure 1A*).* This activity is almost entirely RecA-dependent, as shown in Figure 1B*.* Although supercoiled dsDNA is nicked slowly by Ref in the absence of RecA (top), rapid nicking and subsequent linearization only occur when RecA is present (bottom). Product formation was measured as a percentage of the total DNA in each lane and followed over time (Figure 1C*).* All of the supercoiled dsDNA is nicked quickly, within 20 min after Ref addition. DSBs are formed more slowly, with linear product accumulating over the course of 4 h, and eventually reaching ∼65%. In all figures, unless otherwise noted, the variant RecA E38K was used in place of WT RecA. We have previously demonstrated that RecA E38K enhances the RecA-dependent-targeted DSB activity of Ref beyond that of WT RecA (1)

**Figure 2.**
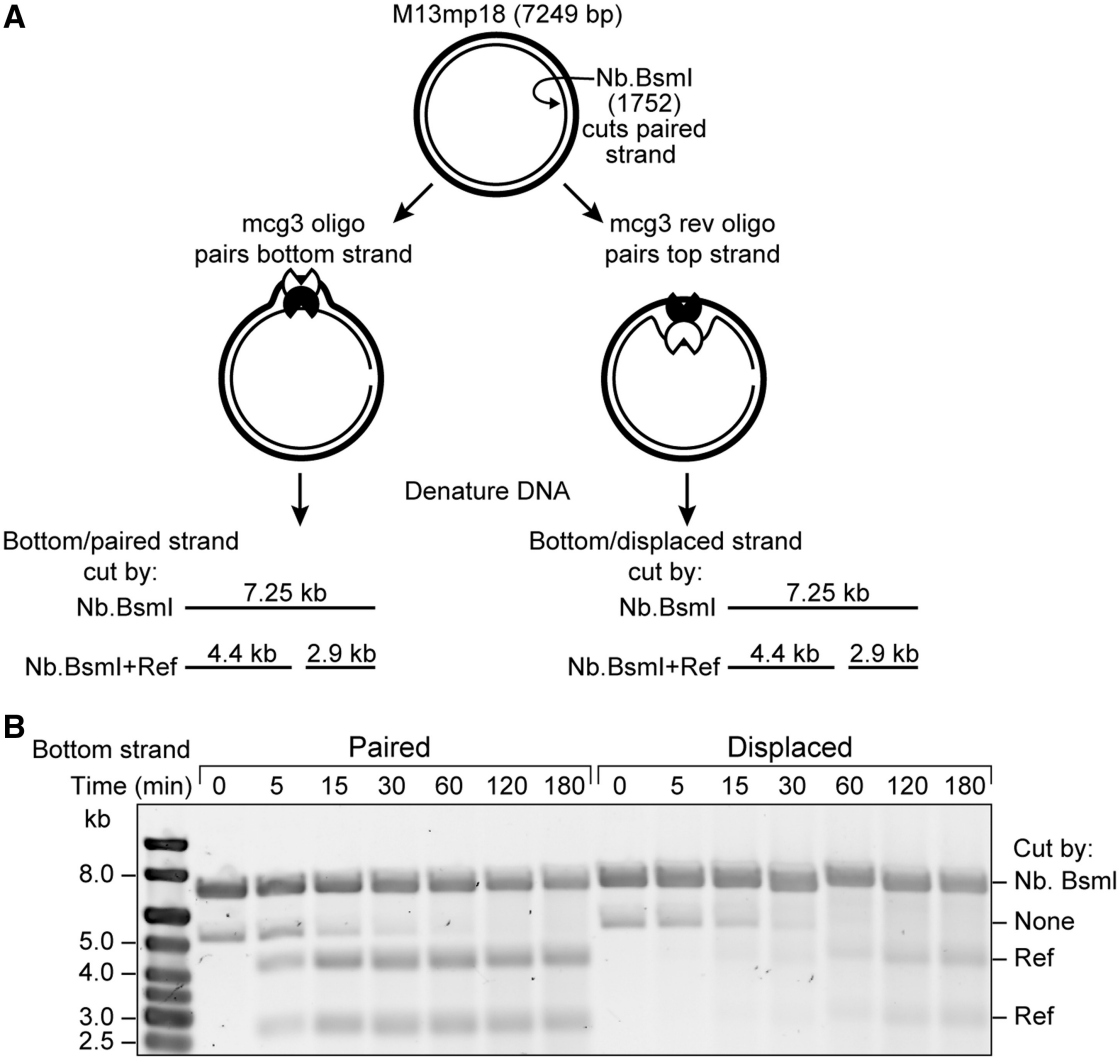
Ref nicks the paired strand of the RecA-mediated D-loop before nicking the displaced strand. (**A**) The reaction scheme is shown. Numbers reference bases from M13mp18 origin. Reaction was carried out as in Figure 1 with the following modifications: M13mp18 nicked dsDNA (8 µMnt) was used instead of cdsDNA. This DNA had been pre-digested with the site-specific nickase Nb.BsmI at position 1752 on the bottom strand (light line). When the mcg3 oligonucleotide was used, the bottom (Nb.BsmI-cut) strand was paired within the D-loop, while the mcg3 rev oligonucleotide resulted in the bottom (Nb.BsmI-cut) strand being displaced within the D-loop. (**B**) Timepoints were removed at the indicated number of minutes after the addition of Ref and stopped with 1× alkaline loading dye. Samples were run on an alkaline agarose gel to visualize the cutting of the bottom strand by Ref.

The D-loop consists of two strands: the strand complementary to the oligonucleotide (the paired strand) and the strand homologous to the oligonucleotide (the displaced strand). To determine which strand of the D-loop Ref cuts first, the targeted nuclease assay was carried out using an M13mp18 cdsDNA substrate that had been nicked on the bottom strand by the site-specific nickase Nb.BsmI (Figure 2A). This reaction did not result in complete nicking, so a small amount of uncut DNA is still visible on the gel. Two reactions were carried out, using the oligonucleotide mcg3 (that pairs with a site on the bottom strand of the duplex in the D-loop) or mcg3rev (that pairs with the same site on the top strand and displaces the bottom strand). After Ref addition, products of either paired (using mcg3) or displaced (using mcg3rev) strand cleavage could be visualized by running the reactions on an alkaline agarose gel to denature the strands. The appearance of products at 4.4 and 2.9 kb indicated RecA-dependent cleavage of the bottom strand by Ref within the D-loop (Figure 2B). Products of paired strand cleavage are apparent only 5 min after the addition of Ref and do not accumulate further after 15 min post-Ref addition (left). Products of displaced strand cleavage only accumulate to a large degree after 60 min of reaction (right). Ref consistently nicks the paired strand of the D-loop first, and the delay in linearization of the duplex is caused by a delay in cutting on the displaced strand.

### Ref cuts the targeting oligonucleotide at preferred sites

**Figure 3.**
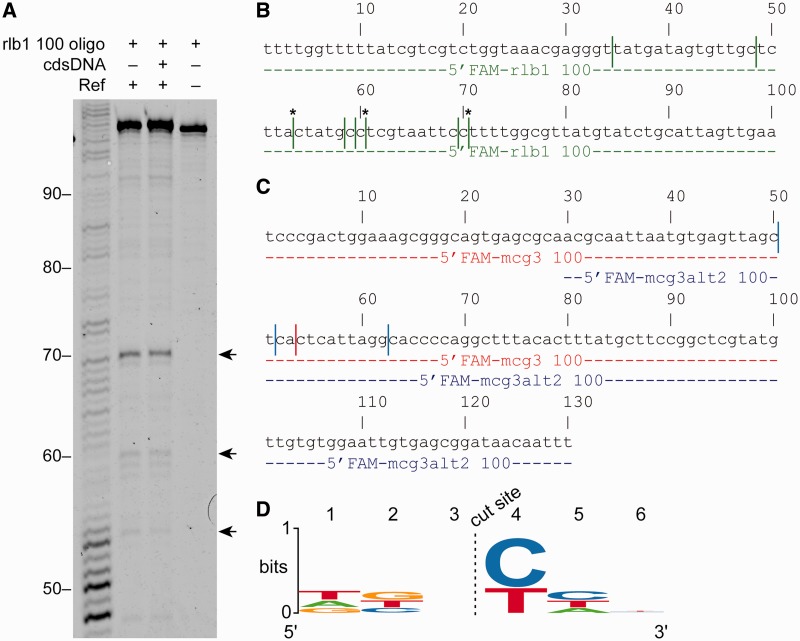
Ref cuts the targeting oligonucleotide at preferred sites upstream of a pyrimidine base. (**A**) Targeted nuclease assays were carried out as described in ‘Materials and Methods’ section except that a 100-mer oligonucleotide that was labeled with 6-carboxyfluorescein on the 5'-end was used instead of the normal 150-mer unlabeled oligonucleotide to create a D-loop in cdsDNA. To size the products of Ref cleavage of the oligonucleotide after 3 h, reactions were run on a 12% sequencing gel. Mock targeted nuclease reactions without cdsDNA were also run, which resulted in some cleavage of the oligonucleotide at the same locations as when cdsDNA is present. Arrows point to the location of products due to cutting at preferred cut sites. (**B**) Location of sites where 5'FAM-rlb1 100 oligonucleotide is cut in (A) are indicated with vertical lines. Asterisks indicate preferred cuts sites, corresponding to arrows in (A)*.* (**C**) Location of 5'FAM-mcg3 100 (red) and 5'FAM-mcg3alt2 100 (blue) cut sites are indicated with vertical lines (gel not shown). (**D**) Sequence logo for cut site location using the 12 cut sites in (B and C)*.* Cut occurs between bases 3 and 4.

To determine whether Ref was also cleaving the targeting oligonucleotide, a targeted nuclease assay was carried out using oligonucleotides labeled on the 5′-end with 6-carboxyfluorescein (5′FAM). Products of the reactions were run on a sequencing gel to determine the precise cut sites on the targeting oligonucleotide (Figure 3A). Using the 5′FAM-labeled rlb1 100-mer oligonucleotide, preferred cut sites on the oligo are located downstream of bases 70, 69 and 53 (shown with arrows in Figure 3A and asterisks in Figure 3B.) There are several other sites that are cut to a lesser degree (indicated with lines in Figure 3B)*.* These same cut sites were preferred when the cdsDNA was eliminated and the targeting oligonucleotide was the only DNA present in the reaction (Figure 3A, left lane). The same experiment was carried out twice more with two different targeting oligonucleotides (gel not shown). These two oligos contained a 70-bp overlap in homology to allow us to determine whether specific sequences were targeted or whether cleavage was directed by some other sequence or structural feature (Figure 3C)*.* Again, Ref cut each targeting oligonucleotide at preferred sites (with fewer sites noted than seen with the oligo used in Figure 3A and B). However, in the region of overlapping homology, the cleavage sites on each of the overlapping oligos did not correspond with each other (compare red cut sites with blue cut sites). All cutting of the targeting oligonucleotide was both RecA- and Ref-dependent (data not shown). The 6-nt region surrounding each cut site (encompassing 3 nt on either side) was compared for the 12 different cut sites identified for the three different targeting oligonucleotides. The 12 sequences were used to generate the sequence logo in Figure 3D*.* No prominent base preference was apparent for five of the six positions. However, a pyrimidine base was always located at position 4, immediately to the 3′-side of the cut site.

### Cutting of the paired and displaced strand exhibit distinct rates and requirements

**Figure 4.**
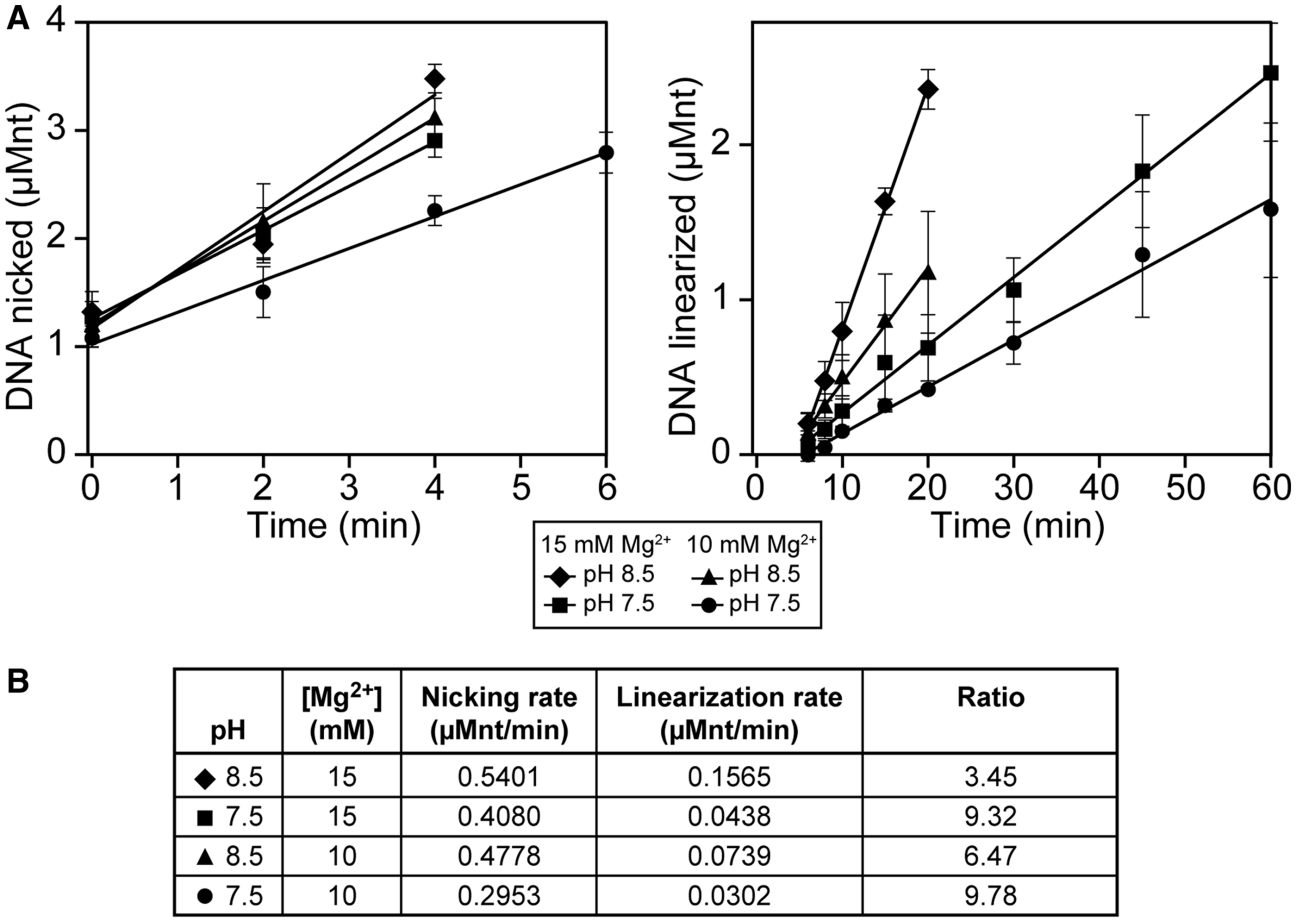
Cutting of the paired and displaced strands exhibit different rates and requirements. (**A**) Targeted nuclease assays were carried out, imaged and analyzed as described in ‘Materials and Methods’ section using buffer A*(pH, Mg). Shown is a graphical representation of gel electrophoresis analysis of products (left: nicked, and right: linear). Filled diamonds correspond to standard buffer conditions used throughout the article. Percentage product formation was converted to actual DNA concentration (in µMnt) and the initial linear portion of each curve was used to calculate the best-fit line and slope. (**B**) Nicking and linearization rates were determined using the slope of the lines in (A). The ratio of the two rates changes with changing buffer conditions, indicating that the nicking and linearization processes are affected differentially by pH and Mg^2+^ concentration.

We wished to determine the extent to which the two separate strand cleavage events, involving the paired strand (cut 1) and the displaced strand (cut 2), were differentially affected by pH and magnesium ion concentration. The targeted nuclease assay was carried out using buffer A*(pH, Mg^2+^), where the pH and magnesium ion concentration (in mM) were varied. DSB generation by Ref occurs optimally in buffer A*(8.5, 15), i.e. at pH 8.5 and 15 mM Mg^2+^. Normal optimized buffer conditions for RecA reactions correspond to a buffer A*(7.5, 10) condition. The targeted nuclease assay was carried out at several different combinations of pH and magnesium ion concentration to determine whether any observed changes in the rates of cut 1 and cut 2 occurred in parallel. The amount of nicked product (the result of paired strand nicking) and linear product (the result of displaced strand nicking) was graphed and best-fit lines were fit to the initial linear portion of each graph (Figure 4A). Rates were determined for nicking and linearization for each buffer condition based on the slope of these lines (Figure 4B). A decrease in pH, Mg^2+^ concentration or both resulted in decreases in both nicking and linearization rates. Additionally, the nicking (cut 1) and linearization (cut 2) rates did not change by the same factors (with respect to each other) in each alteration of conditions. At the optimal reaction conditions of pH 8.5 and 15 mM of Mg^2+^, the ratio of the two rates was 3.45. When pH was reduced to 7.5, Mg^2+^ concentration was reduced to 10 mM or both, the ratio of the two rates increased to 9.32, 6.47 and 9.78, respectively. Cleavage of the paired strand was more sensitive to changes in pH than changes in magnesium: reduction of the pH results in a 24% decrease in reaction rate, whereas reduction of magnesium results in only a 12% decrease in reaction rate. The rates of cut 2 exhibited greater changes with respect to these alterations in conditions. Reduction in pH results in a 72% decrease in reaction rate, whereas reduction of magnesium ion concentration results in a 53% decrease in reaction rate. The changes in pH and magnesium ion requirements for the two cleavage events could reflect potential changes in protein and/or DNA structure. Alternatively, they could reflect more substantive differences in chemical mechanism. At a minimum, they reinforce the differential activity of Ref protein on the two DNA strand substrates.

### The variant Ref H153A nicks the D-loop, but cannot create DSBs

**Figure 5.**
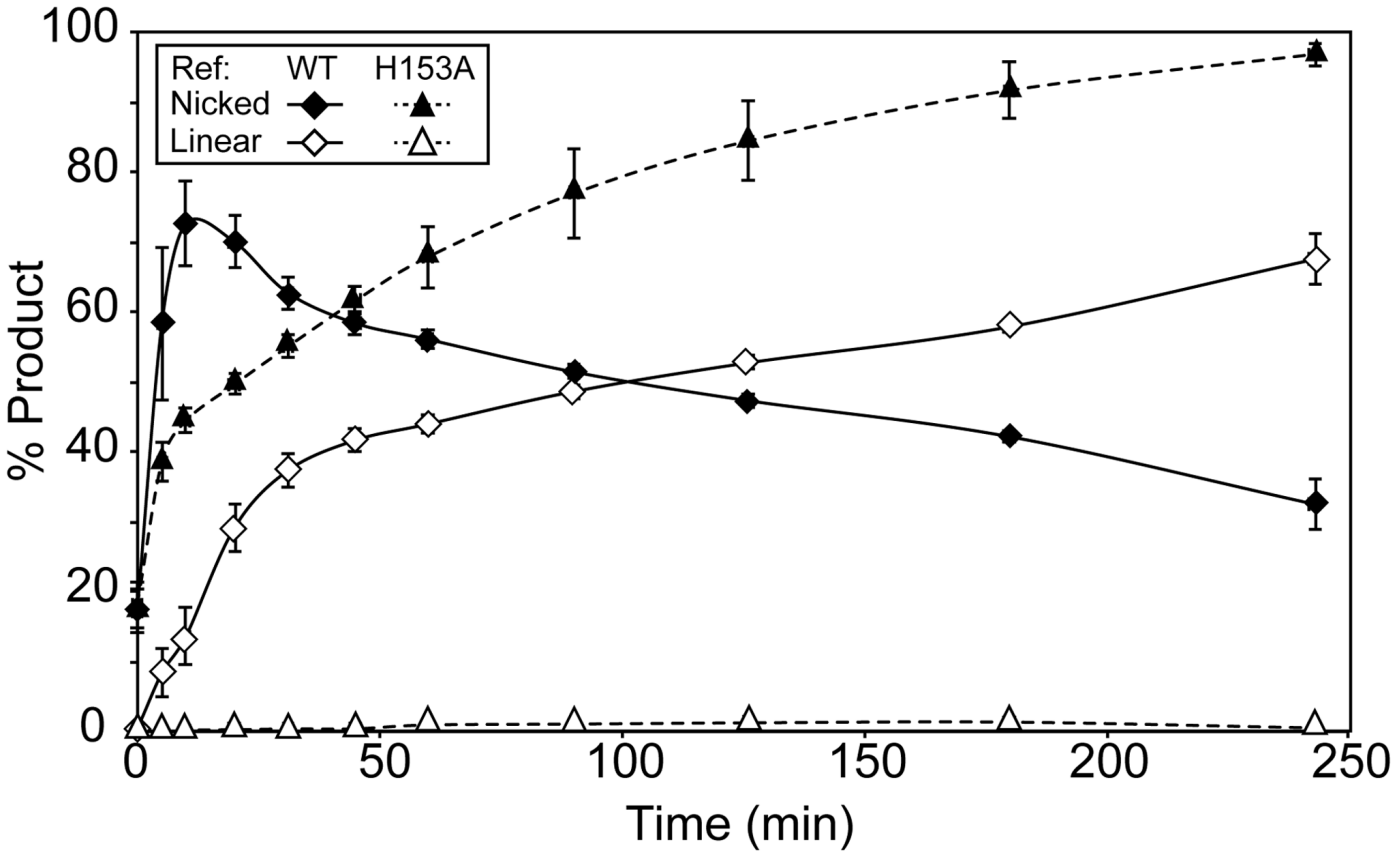
Ref H153A nicks within the D-loop, but does not create DSBs. The targeted nuclease assay was carried out as in Figure 1. Shown is a graphical representation of gel electrophoresis analysis of the cleavage products. An average of three independent trials is reported. Ref H153A does not create any substantial amount of linear DNA. Diamonds: WT Ref, triangles: Ref H153A. Filled: nicked product, open: linear product.

Previously, we reported that the Ref H153A variant cannot create targeted DSBs or degrade cssDNA. Thus, we reported a loss of nuclease activity because of this single mutation of a putative active site residue (1). However, changes to the experimental protocol allowed us to separately track the two cuts leading to a targeted DSB. Ref H153A carried out cut 1, nicking the duplex within the D-loop, a process that could not be detected in the earlier study. However, it did not carry out cut 2 (Figure 5). Experiments similar in design to those shown in Figure 2 confirmed that Ref H153A cleaved only the paired DNA strand in the D-loop, and left the displaced strand intact (data not shown). Ref H153A carried out cut 1 more slowly than WT Ref (compare filled triangles with filled diamonds, respectively), but nicked the paired strand of the duplex nearly to completion in 4 h. Over the course of these 4 h, accumulation of linear product (as a result of cut 2) was minimal (open triangles).

### For targeted DSB generation by Ref, the ATP hydrolysis activity of RecA filament is critical for cut 2 but not for cut 1

**Figure 6.**
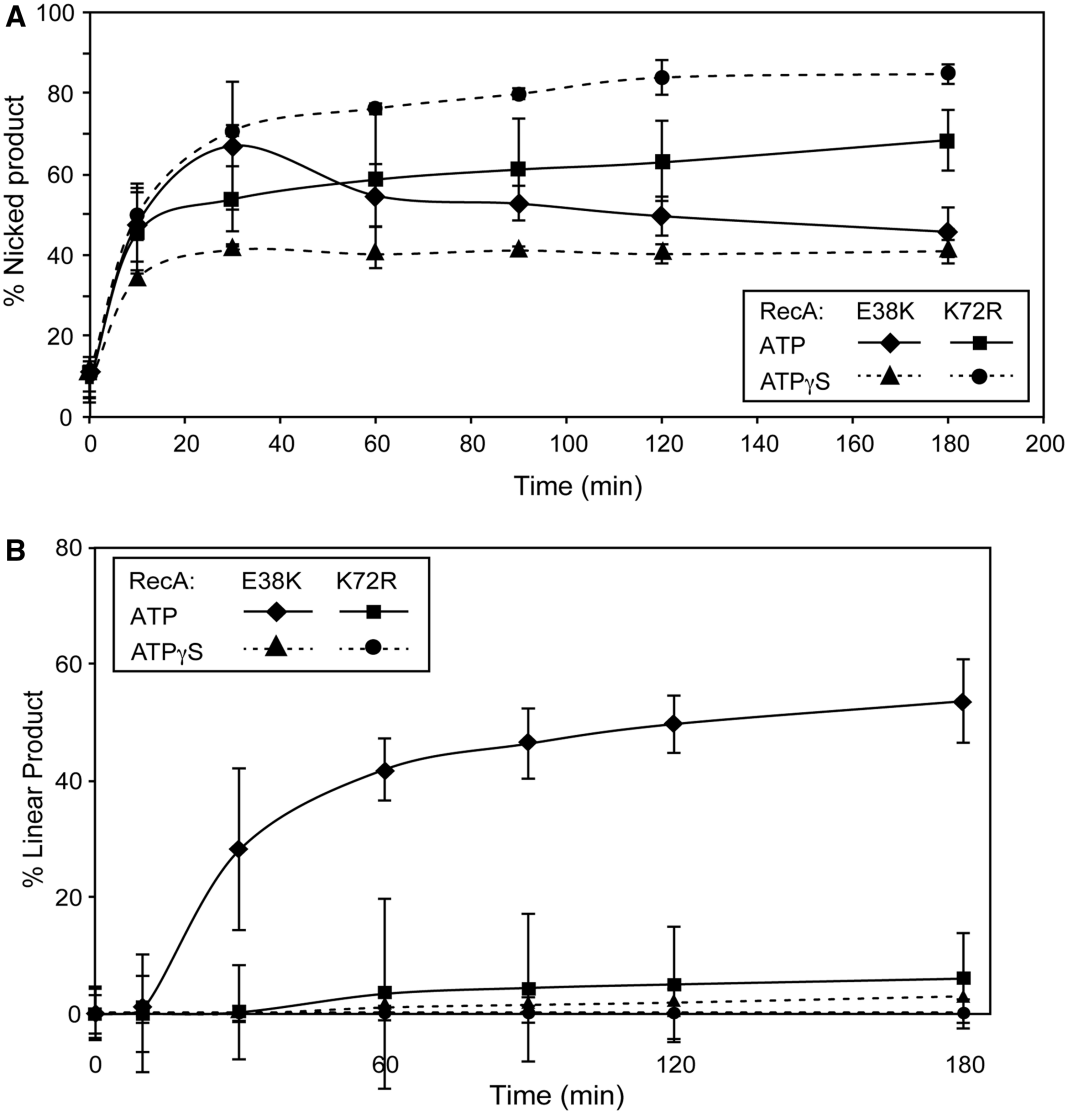
Targeted DSB formation by Ref requires a RecA filament that is actively hydrolyzing ATP. The targeted nuclease assay was carried out as stated in ‘Materials and Methods’ section with RecA K72R or ATPγS substituted for RecA E38K or ATP, respectively, as indicated. (**A**) Appearance of nicked product (cut 1) over time as a percentage of total DNA in each lane of the agarose gel. An average of three independent trials is reported. (**B**) The appearance of linear product (cut 2) over time as a percentage of total DNA in each lane of the agarose gel. An average of three independent trials is reported.

To investigate how the function of ATP hydrolysis by the RecA nucleoprotein filament affects targeted DSB generation by Ref, a targeted nuclease reaction was carried out under conditions that prevent ATP hydrolysis by the RecA filament (Figure 6). RecA K72R was used in place of RecA E38K. This variant contains a mutation in the Walker A box, preventing ATP hydrolysis (17). Also, ATPγS, a non-hydrolyzable ATP analog, was used in place of ATP. Additionally, RecA K72R and ATPγS were used in combination. Nicking of the supercoiled duplex target (cut 1) was examined first. In all three ATP hydrolysis-null conditions (RecA K72R, ATPγS or the two in combination), Ref generated nicked circular duplexes (Figure 6A). Ref nicked only the paired strand of the D-loop in all cases (data not shown). The extent and rate of the cut 1 reaction varied among the three ATPase-constrained conditions. RecA E38K in combination with ATPγS resulted in ∼40% of the dsDNA being nicked within the D-loop, all occurring in the first hour of the reaction. Use of RecA K72R in combination with ATP resulted in >60% of the dsDNA being nicked. RecA K72R in combination with ATPγS resulted in >80% of the dsDNA being nicked within 3 h. In both cases, the amount of nicked DNA continued to climb throughout the reaction. Previous research has demonstrated the differences in RecA filament architecture and dynamics when different methods are used to prevent ATP hydrolysis (12,17–19), and these differences clearly affect the ability of Ref to cut the paired DNA strand within the D-loop.

When ATP hydrolysis was prevented, few targeted DSBs were created by Ref under any conditions (Figure 6B). In the control reaction using RecA E38K and ATP, ∼60% of the cdsDNA was linearized over 3 h. RecA-mediated ATP hydrolysis is thus required for the second cleavage event (cut 2) needed to generate a Ref-mediated double-strand break at a D-loop, but not for cut 1.

## DISCUSSION

The HNH endonuclease Ref will cleave both strands of a target duplex at the site of a RecA-generated D-loop. In the present study, we demonstrate that the cleavage of the two strands occurs in a prescribed order, and that the two steps exhibit different rates and requirements. The strand directly paired with the oligonucleotide used to create the D-loop is cleaved first. Cut 1 is relatively fast, can be promoted by the Ref active site mutant H153A and does not require hydrolysis of ATP by RecA protein. The displaced strand is also cleaved, but much more slowly. Cut 2 is much slower, is not promoted by the H153A mutant of Ref and requires RecA-mediated ATP hydrolysis. The two cleavage events are also affected differently by changes in pH and magnesium ion concentration. It is not clear whether single or multiple cuts occur on each strand of any given D-loop, although there are definitely multiple preferred cut sites.

The rate differences between cut 1 and cut 2 (Figures 1 and 2) may be due to the accessibility of the paired and displaced strands within the D-loop to the Ref protein. Crystallographic and single-molecule experiments show that the displaced strand of the D-loop is bound within the secondary binding site on the RecA filament. As a result, the phosphate backbone of this strand is buried within the filament. In contrast, the paired strand of the D-loop makes minimal contacts with the RecA filament and is held in place primarily by its Watson–Crick base pairing to the targeting oligonucleotide (20–23). Thus, Ref appears to have more access to the phosphate backbone of the paired strand DNA, resulting in a faster rate of cleavage of that strand. Cut 2 within the D-loop requires ATP hydrolysis, and the cleavage of RecA-bound cssDNA is greatly enhanced by ATP hydrolysis. Ref H153A is incapable of cleavage in both scenarios. As cssDNA would be bound in site 1 of the RecA filament and similarly buried as the displaced strand is in site 2 during targeted cleavage, the ‘limited access’ hypothesis is further supported.

In this study, we have also observed Ref-mediated cleavage of the targeting oligonucleotide (Figure 3). This cleavage occurs slowly, with <10% of the oligo being cleaved after 180 min of reaction. Given that similar amounts and patterns of cleavage are seen in the presence and absence of cdsDNA, this cutting likely occurs outside of the D-loop structure. The reactions contain enough RecA to coat all of the targeting oligos present in the reaction, but the amount of RecA nucleoprotein filaments formed far exceeds the number of potential D-loop sites in these reactions. We have previously demonstrated that Ref will cleave ssDNA bound by a RecA filament (1). Thus, the fact that Ref will cleave the targeting oligonucleotide is not surprising and may be facilitated by extension and underwinding of the DNA within the primary binding site of the RecA filament. (20) A preference for oligo cleavage adjacent to pyrimidine nucleotides represents a new observation for Ref-mediated cleavage of RecA-bound ssDNA. We previously demonstrated preferred cleavage sites within the D-loop on both the paired and displaced strands, but here cleavage tended to occur adjacent to A or T nucleotides (1). For both the paired and displaced strands, there is a pronounced tendency to cleave on the 5′-side of thymidine. Notably, this is the cleavage preference exhibited by the HNH endonuclease colicin E9 (24).

The differing effects of pH and magnesium ion concentration (Figure 4) may eventually provide additional mechanistic clues for the two Ref-mediated cleavage steps. When the pH is reduced from 8.5 to 7.5 and magnesium ion concentration remains constant, the cut 1/cut 2 rate ratio changes from 3.45 to 9.32. The rate of cut 2 declines much more than the rate of cut 1 when the pH is reduced. The same trend is seen (although to a lesser degree) when the pH remains constant at 8.5 but the magnesium ion concentration drops from 15 to 10 mM. Magnesium ion is used by RecA in at least two ways. As is the case for all ATPases, the substrate for ATP hydrolysis is a Mg–ATP complex. Magnesium ion in excess of the ATP concentration modulates an inhibitory effect of the RecA C-terminus (25). Magnesium ion may also be used by Ref in the active site. There is precedence in the literature for HNH endonucleases using different divalent cations in their active sites, leading to significant changes in the nicking versus double-strand break activity profiles due to differential chelation of the ions. (24,26–28) The cut 1 and cut 2 reactions occur over a pH range that could readily reflect the ionization of His residues. Although there is only one His residue in a RecA monomer, we note that there are multiple His residues located near and within the Ref active site that may be affected by the pH changes (1).

In particular, histidine 153 is located within the putative active site, and chelates a zinc atom that is stably bound within the structure. Previously, we reported that mutating this residue to an alanine abolished all nuclease activity, while leaving the bound state of the zinc ion unchanged (1). Here, we have shown that Ref H153A is only deficient in carrying out cut 2, cleavage of the displaced strand of the D-loop (Figure 5). Cut 1, a process that we were not able to monitor in the previous study due to the design of the assays used, still proceeds, albeit more slowly than is seen with WT Ref. It is possible that cut 2 is promoted at a different active site pocket within Ref. More likely, some aspect of RecA filament dynamics affects the geometry of hydrolytic phosphodiester bond cleavage within the single proposed active site, such that histidine 153 is a catalytic residue only for cleavage of the displaced strand.

His 153 occupies a structural position analogous to His 131 in the HNH endonuclease colicin E9 (29,30). His 131 has an unknown role in the cleavage of DNA by colicin E9, but the authors suggest that it may bind the DNA backbone (24). Colicin E9 exhibits DNase activity with several different divalent and transition metal ions in the active site, notably magnesium, nickel, cobalt and manganese (28). However, DNase activity is not seen when zinc is in the active site, due to coordination of the metal by His 131, which is not seen with other metals (27). Colicin E9 H131A still bound to zinc and nicked plasmid DNA in the presence of nickel, but lost its nicking activity in the presence of magnesium (26). Different cleavage mechanisms are proposed depending on whether nickel or magnesium is bound in the active site (24).

It is possible that Ref exhibits similar mechanistic-dependence on metal ion identity. As RecA requires magnesium, it would be impossible to perform similar tests looking at Ref activity in the presence of single metals. Individual monomers of Ref could use either the bound zinc ion, which His 153 will coordinate. Alternatively, Ref could use a magnesium ion from the buffer, which His 153 will probably not coordinate based on structural similarities with colicin E9 (27). The zinc-bound version may only be required for displaced strand cleavage, either for access to the strand while buried in the secondary binding site of the RecA filament, or for actual catalysis. Ref H153A may still be able to bind magnesium and would be able to use this form of the enzyme to cleave the paired strand. Duplex cleavage in two mechanistically distinct steps by a single active site would not be unique to the Ref nuclease. This pattern has been shown before with other nicking endonucleases (NEases), such as I-BmoI, a GIY-YIG homing endonuclease that also creates DSBs through two independent nicking reactions (31).

The requirement for RecA-mediated ATP hydrolysis for cut 2, but not for cut 1, along with the much-reduced rate of cut 2, suggests that the limiting factor for cut 2 is some property of the RecA filament rather than an activity of Ref. RecA-mediated ATP hydrolysis has several effects on RecA, but a primary effect is on disassembly of RecA filaments (32–39). Cut 2 may occur only at the disassembling end of a RecA filament, and require ATP hydrolysis to create such an end with sufficient proximity to cut 1. This is supported by the observation that the total amount of DNA nicked within the D-loop (cut 1) changes substantially, depending on the method of preventing ATP hydrolysis. RecA K72R creates short gapped filaments, whereas RecA E38K in the presence of ATPγS creates longer filaments. In both cases, these filaments are capable of forming D-loops, but are incapable of disassembling. However, RecA K72R (with ATP or ATPγS) filaments have many more ends from which Ref can work to create cut 1, resulting in an increase in cut 1 rate when compared with RecA E38K with ATPγS.

**Figure 7.**
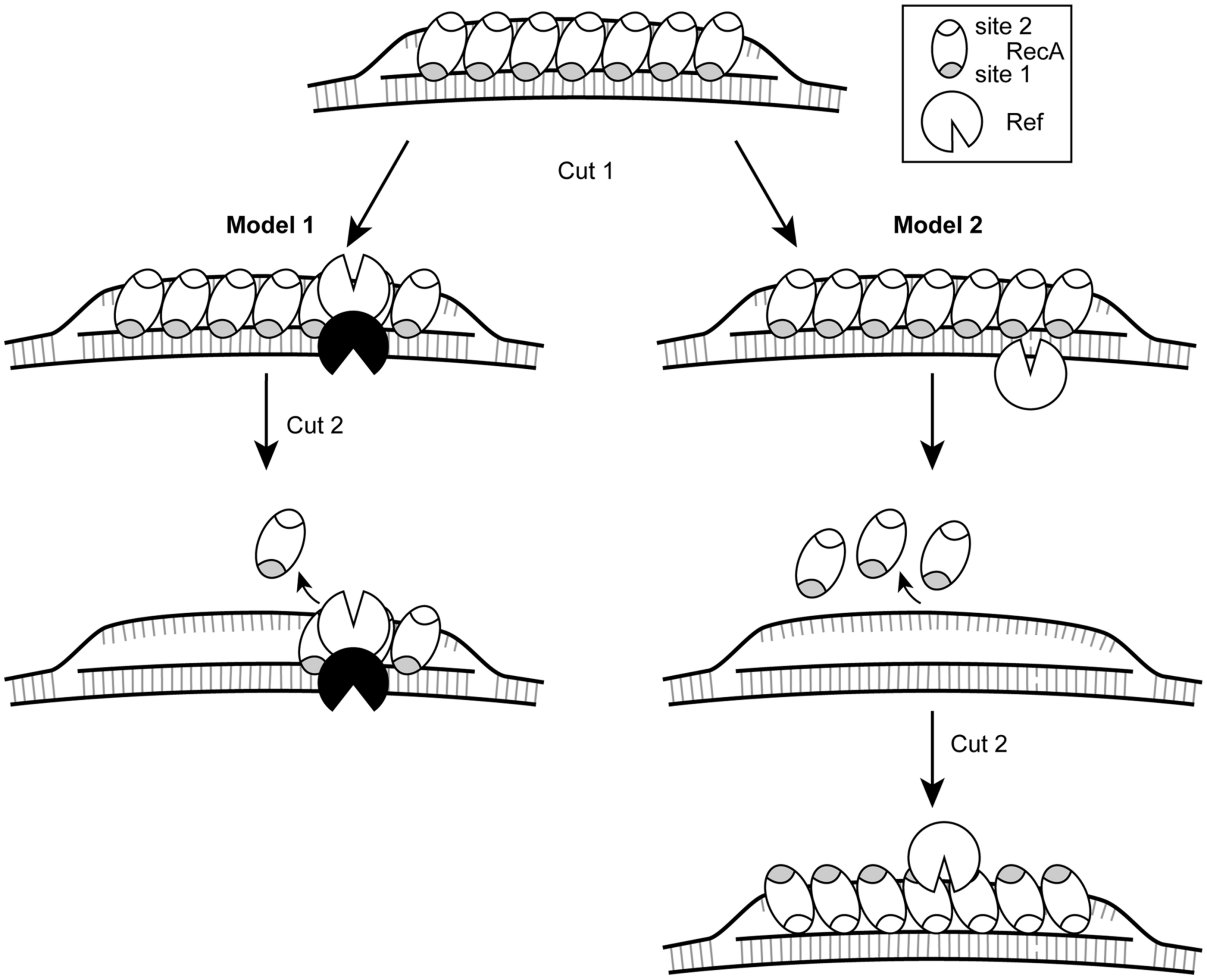
Two models for the creation of targeted DSBs by Ref. In both models, RecA creates a D-loop by binding the targeting oligonucleotide in the primary binding site (gray), and after finding homology, binds the displaced strand in the secondary site (white). Model 1 (left): a dimer of Ref nicks the paired strand of the D-loop (cut 1). ATP hydrolysis-dependent disassembly of the RecA filament occurs in the 5'–3' direction. When filament disassembly occurs at the site of Ref binding, Ref can then access the displaced strand to create cut 2. Model 2 (right): a Ref monomer nicks the paired strand of the D-loop, followed by total ATP hydrolysis-dependent disassembly of the RecA filament, but leaving the D-loop DNA structure intact. The RecA filament then reassembles with the displaced strand (which has single-stranded character) in the primary binding site, and Ref cleaves the displaced strand.

Combining the above observations produces Model 1, or the ‘limited access’ model (Figure 7, left). In this model, RecA binds the targeting oligonucleotide in the filament primary site and forms the D-loop by binding the displaced strand in the filament secondary site. The paired strand is held by the filament via its Watson–Crick base pairs with the oligo. This results in the phosphate backbone being openly accessible to a dimer of Ref (Gruber,A. and Cox,M., manuscript in preparation), and Ref cleaves the paired strand to generate cut 1. Owing to the buried nature of the displaced strand within the RecA filament, the Ref dimer must wait for ATP hydrolysis-mediated disassembly of the RecA filament to occur in proximity to the bound Ref dimer. When this occurs, the second monomer of Ref will cleave the displaced strand possibly using a distinct chemical mechanism to create a DSB.

An additional possibility is reflected in Model 2, or the ‘complete disassembly’ model (Figure 7, right). Here, the D-loop forms as in model 1, with the targeting oligonucleotide in the primary site of the RecA filament and the displaced strand in the secondary site. A Ref monomer cleaves the paired strand because of the easy accessibility of the phosphate backbone. Then, the entire RecA filament must disassemble in an ATP hydrolysis-dependent manner, leaving the displaced strand unpaired. The RecA filament reassembles with the displaced strand in the primary binding site and the targeting oligonucleotide in the secondary site, creating an inverted D-loop. Then, another monomer of Ref would bind and cleave the originally displaced strand (which is now paired). This model would require that the three-stranded D-loop structure remain stable after the dissociation of the RecA filament, which would be assisted by the relaxation of negative supercoiling following cut 1 by Ref. Further experimentation will allow us to refine our model system.

The long-term goal of this work is to understand the mechanistic function of the Ref protein, in particular, the RecA-dependent targeted nuclease activity, such that this Ref/RecA system can be exploited for applications in biotechnology. Recently, much work has been focused on using the CRISPR/Cas9 system derived from type II prokaryotic CRISPR systems for creating targeted DSBs in a variety of organisms including bacteria (40), yeast (41), *Caenorhabditis elegans* (42), *Drosophila* (43), zebrafish (44), mice (45) and human cell lines (46). These systems use an engineered small guide RNA, which contains a 20–40-nt spacer sequence identical to the genomic sequence to be cut (47). The Cas9 protein forms a complex with this small guide RNA and the complex invades the genomic DNA duplex at the homologous sequence, after which Cas9 cleaves the two strands of the duplex using two different active sites. The HNH-like domain cleaves the paired strand of the R-loop, while the RuvC-like domain cleaves the displaced strand (48). If exogenous DNA with the desired gene sequence is provided, homologous recombination repair of the DSB generated by Cas9 will cause the original genetic sequence to be replaced with the desired sequence (47). This system is promising, with the caveat that a high rate of off-target cleavage and mutation events has been seen in some applications (49).

The Ref/RecA system has a somewhat greater complexity than CRISPR/Cas9. The two proteins needed are Ref and the RecA proteins, and a targeting oligonucleotide is also required. For effective genome targeting in organisms beyond bacteria, these three components must likely be somehow tethered. Efforts to effect such tethering may be profitable, as the single ssDNA oligo required does not have any sequence specificity such as the protospacer adjacent motif (PAM) in the sequence needed for the Cas9 editing system. The PAM requirement only allows ∼40% of a genome to be targeted by Cas9 (47). Owing to the requirement for a longer targeting sequence (100–150 nt is optimal), our system may also exhibit enhanced specificity over the Cas9 system, which allows only 20–40 nt of homology matching (48). Finally, as RecA is required for Ref to create targeted DSBs, RecA would already be concentrated near the site of the DSB. This may allow for a higher rate of incorporation of the exogenous DNA through homologous recombination. Currently, our laboratory is directly investigating the use of Ref for creating targeted DSBs and incorporation of mutated gene sequences *in vivo* in bacteria.

Although there is no distinct evolutionary role known for the Ref protein and its effect on the life cycle of bacteriophage P1, we are pursuing two working hypotheses. First, preliminary data from our laboratory indicate that *ref* selectively inhibits the lysogenic life cycle of the phage. Ref will create DNA damage (DSBs or nicks) in the presence of RecA, which is increasingly produced during the bacterial SOS response. Ref may thus encourage lysis of the bacterial cell under conditions of stress by using the cell’s own RecA-mediated repair processes against it.

A second hypothesis is inspired by some evident parallels between the Ref/RecA system and the type I-E CRISPR system found in *E. coli.* This class of CRISPR system contains a multi-subunit protein complex, Cascade, which binds to and matures the pre-crRNA into CRISPR RNA (crRNA), and the helicase/nuclease Cas3, which unwinds and degrades the target DNA in an ATP-dependent manner (50,51). The Cascade complex is made up of CasAB_2_C_6_DE, with the six CasC monomers forming a helical nucleoprotein filament surrounding the single-stranded spacer region of the crRNA (52). This complex is similar in structure to the RecA filaments formed on ssDNA (20,53). Ref may recognize the Cascade/crRNA complex and degrade it before it can invade the target sequence on the bacteriophage genome, preventing the CRISPR system from targeting the phage for degradation. Another possibility for Ref involvement with the host CRISPR system may involve the ability of Ref to enhance RecA-mediated microhomologous recombination (3). The presence of Ref could cause RecA to recombine at the repeated regions in the CRISPR loci to eliminate spacer sequences, making the host more generally susceptible to phage infection. The interaction of the phage proteins with the bacterial CRISPR system is not unprecedented: at least five genes from phages that infect *Pseudomonas aeruginosa* were identified as anti-CRISPR genes in a genetic screen (54). Continuing work will be directed at identifying the *in vivo* utility of the Ref nuclease for bacteriophage P1.

## FUNDING

National Institutes of Health [R01 GM32335 to M.C., T32 GM07215 to E.R.] and the National Science Foundation [DGE-1256259 to E.R.]. Funding for open access charge: National Institutes of Health [GM32335].

*Conflicts of Interest*: Erin Ronayne has no conflicts to declare. Michael M. Cox is a board member, consultant and shareholder of Recombitech, Inc. His relationship to the company is managed by the University of Wisconsin–Madison in accordance with its conflict of interest policies.
